# Human aldehyde oxidase (hAOX1): structure determination of the Moco‐free form of the natural variant G1269R and biophysical studies of single nucleotide polymorphisms

**DOI:** 10.1002/2211-5463.12617

**Published:** 2019-04-15

**Authors:** Cristiano Mota, Mariam Esmaeeli, Catarina Coelho, Teresa Santos‐Silva, Martin Wolff, Alessandro Foti, Silke Leimkühler, Maria João Romão

**Affiliations:** ^1^ UCIBIO Departamento de Química Faculdade de Ciências e Tecnologia Universidade Nova de Lisboa Caparica Portugal; ^2^ Department of Molecular Enzymology Institute of Biochemistry and Biology University of Potsdam Germany; ^3^ Department of Physical Biochemistry Institute of Biochemistry and Biology University of Potsdam Germany

**Keywords:** human aldehyde oxidase, molybdenum cofactor, single nucleotide polymorphism, xanthine oxidase

## Abstract

Human aldehyde oxidase (hAOX1) is a molybdenum enzyme with high toxicological importance, but its physiological role is still unknown. hAOX1 metabolizes different classes of xenobiotics and is one of the main drug‐metabolizing enzymes in the liver, along with cytochrome P450. hAOX1 oxidizes and inactivates a large number of drug molecules and has been responsible for the failure of several phase I clinical trials. The interindividual variability of drug‐metabolizing enzymes caused by single nucleotide polymorphisms (SNPs) is highly relevant in pharmaceutical treatments. In this study, we present the crystal structure of the inactive variant G1269R, revealing the first structure of a molybdenum cofactor (Moco)‐free form of hAOX1. These data allowed to model, for the first time, the flexible Gate 1 that controls access to the active site. Furthermore, we inspected the thermostability of wild‐type hAOX1 and hAOX1 with various SNPs (L438V, R1231H, G1269R or S1271L) by CD spectroscopy and ThermoFAD, revealing that amino acid exchanges close to the Moco site can impact protein stability up to 10 °C. These results correlated with biochemical and structural data and enhance our understanding of hAOX1 and the effect of SNPs in the gene encoding this enzyme in the human population.

**Enzymes:**

Aldehyde oxidase (EC1.2.3.1); xanthine dehydrogenase (EC1.17.1.4); xanthine oxidase (EC1.1.3.2).

**Databases:**

Structural data are available in the Protein Data Bank under the accession number 6Q6Q.

**Abbreviations**
AOX: aldehyde oxidase
ESRF: European Synchrotron Radiation Facility
hAOX: human aldehyde oxidase
Moco: molybdenum cofactor
PDB: Protein Data Bank
PTSD: protein thermal shift dye
SNP: single nucleotide polymorphism
*T*_m_: melting temperature
WT: wild‐type
XDH: xanthine dehydrogenase
XOR: xanthine oxidoreductase
XO: xanthine oxidase

The human aldehyde oxidase (hAOX1) is an important enzyme involved in the liver metabolic conversion of drugs and xenobiotics compounds 1, 2, 3, 4. The relevance of aldehyde oxidases (AOXs) in drug metabolism has been uncovered in the last decade and has attracted huge interest from several pharmaceutical companies, particularly due to implications of unpredicted metabolism in phase I clinical trials 5, 6. In addition, the chemical strategies used by drug design companies to reduce cytochrome P450‐mediated metabolic clearance in prospective therapeutic agents led to new drug molecules that are preferred substrates to AOX 7, raising additional interest in this group of proteins.

Human aldehyde oxidase 1 is a homodimeric molybdenum cofactor (Moco)‐containing protein with a molecular mass of 300 kDa (2 × 150 kDa) that belongs to the xanthine oxidase (XO) family of mononuclear molybdenum enzymes 8, 9. Each subunit contains one Moco and two spectroscopically distinct [2Fe–2S] clusters (Fe/SI and Fe/SII) and one FAD cofactor. These centres are, respectively, located in the C‐terminal (85 kDa), N‐terminal (20 kDa) and intermediate (40 kDa) domains of the protein 10. AOXs share a high degree of structural similarity with xanthine oxidoreductases (XOR) 11 and major differences regarding the substrate specificity and the existence of two interconvertible forms in XOR [XO and xanthine dehydrogenase (XDH)] 12. XOR is the key enzyme in the catabolism of purines, oxidizing hypoxanthine to xanthine, and xanthine to uric acid, at the molybdenum centre, by hydroxylation with the oxygen atom derived from water. The reducing equivalents thus introduced into Moco are rapidly transferred via Fe/SI and Fe/SII, to FAD, where the reduction of NAD^+^ (XDH form) or of oxygen (XO form) occurs 13. In contrast to XOR, the substrate specificity of AOXs is very broad performing diverse reactions that include oxidations (e.g. aldehydes and aza‐heterocycles), hydrolysis of amide bonds, and reduction reactions (e.g. nitro, *S*‐oxides and *N*‐oxides reductions) 4. On the other hand, molecular oxygen is the unique AOX electron acceptor known so far. The crystal structures of hAOX1 in the substrate‐free form [Protein Data Bank (PDB) ID: 4UHW] and in complex with the substrate phthalazine and the noncompetitive inhibitor thioridazine (PDB ID: 4UHX) are known and contributed with novel and important structural features to the clarification of the role of hAOX1 in drug metabolism 10. However, despite the extensive ongoing investigations on the role of AOXs in drug metabolism, there are only very few studies reporting the stability of enzymes from this family not taking into account structural aspects 14.

One of the critical issues faced by pharmaceutical companies is the identification and characterization of natural variants of the enzymes that impact drug metabolism and, thus, have to be considered for the effectiveness and toxicity in personalized therapies. In the human population, a major source of interindividual variability in hAOX1 enzymatic activity arises from single nucleotide polymorphisms (SNPs). Recent studies demonstrated that some of these missense SNPs affect the catalytic function of the enzyme in either a positive or a negative fashion 15, 16, 17. The first crystal structure of a hAOX1 SNP to be reported was the S1271L, where, apart from some catalytic differences, no major structural changes were observed when compared to the wild‐type (WT) enzyme 16. In the current study, we crystallized and solved the 3D structure of the natural, inactive, G1269R variant, where the cofactor is completely absent, which constitutes the first crystal structure of a Moco‐free form of AOX. In addition, we report the first biophysical study integrating CD spectroscopy and ThermoFAD to compare the protein stability of four hAOX1 SNPs (L438V, R1231H, G1269R and S1271L) with that of the WT enzyme.

## Results and Discussion

### Crystal structure of hAOX1‐G1269R

**Figure 1:**
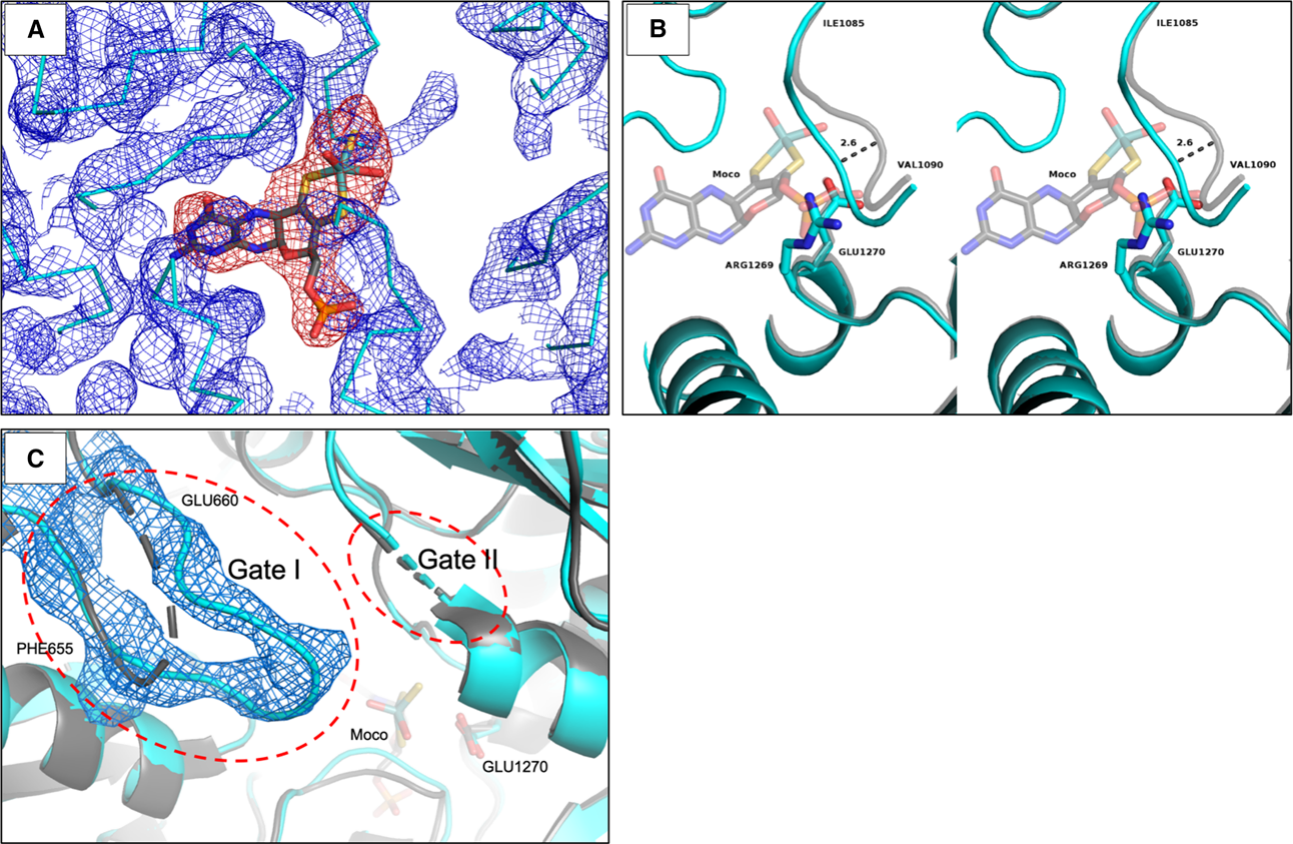
Selected representations of the hAOX1‐G1269R crystal structure (cyan) superimposed with the hAOX1 WT structure (grey): (A) attempt to model the Moco cofactor: 2mFo‐DFc electron density maps represented in blue with contour level (σ) of 1.0 and negative mFo‐DFc electron density maps represented in red with contour level (σ) of −3.0; (B) stereo view of the enzyme active site from both hAOX1‐G1269R (cyan) and hAOX1 WT (grey); and (C) representation of the 2Fo‐Fc electron density map for the region of the hAOX1‐G1269R Gate I (residues Phe655‐Glu660; electron density contoured at σ 1.0).

The hAOX1‐G1269R variant has been previously reported as an inactive form of the enzyme 16. Gly1269 is adjacent to Glu1270, which acts as a catalytic base initiating the first step of the substrate hydroxylation mechanism 18, 19, and its replacement by a bulky and charged residue (Arg) is very likely to influence the enzyme activity. To understand the effect of this amino acid exchange from a structural point of view, we crystallized the variant and collected diffraction data up to 3.1 Å resolution. The structure was determined by molecular replacement using the substrate‐free hAOX (herein designated as WT) structure as a search model (PDB ID: 4UHW). The overall fold of G1269R is very similar to the WT 10 with a RMSD of 0.321 Å for 949 C‐alphas, indicating that the structural integrity of the protein is preserved. In spite of the similarities, the G1269R crystal structure is the first structure of hAOX1 obtained without the entire Moco cofactor. Evidence for the absence of Moco in the crystal structure of this natural variant is shown by negative electron density maps when trying to model the cofactor, even with partial occupancy (Fig. 1A). This Moco‐free form of the enzyme agrees with our previous biochemical analysis that indicated no activity towards phenanthridine oxidation, and only 31% of Mo incorporation 16. Since a saturation of 5% of Moco in the enzyme was determined (data not shown), this reveals unspecific molybdate binding to the enzyme and conclusively only the Moco‐free form of the enzyme crystallized. When comparing the refined models of the WT hAOX1 and G1269R, we identified major differences at the oxidation site (Mo active site), where the Arg1269 side chain is placed at the Moco pocket, occupying the Moco's phosphate position in the WT enzyme (Fig. 1B). In this variant, Arg1269 clearly prevents the incorporation of the Moco cofactor, by blocking the cavity with its positively charged guanidinium side chain, which is within hydrogen bonding distance of the carbonyl oxygens of Ser1089 and Gly1088 (not shown). The Ser1086‐Gly‐Gly‐Ser1089 loop close to the Moco in the WT enzyme appears 2.6 Å shifted towards the vacant pocket in this variant (Fig. 1B). Remarkably, despite the lack of Moco, the amino acid residues involved in the stabilization of the pterin moiety (Gln113, Gln776, Phe807, Arg921 and Gln1203) remain in the same orientation as in all Moco‐containing hAOX1 structures solved so far. In the Moco‐free (reported by the authors as ‘demolybdo’) form of mouse liver XOR 20, 21, the position of the side chains of the Moco‐surrounding residues is also preserved by a complex hydrogen bond network and no major differences are found when comparing, for example, the bovine milk XDH (PDB ID: 3UNC) 22 with the Moco‐free form of rat liver XOR (PDB ID: 4YRW) 21.

An interesting feature of the G1269R crystal structure is that Gate 1 (Phe655‐Glu660) is well ordered and shows clear electron density that enabled modelling the main chain atoms of the loop for the first time (Fig. 1C). This Gate 1 is located at the entrance of the catalytic pocket, and we have previously proposed that it might be involved in substrate sequestration and orientation 10. Gate 2, however, is disordered in this variant (Leu880‐Ser883), and, as for the WT protein, it was not possible to model the two acidic amino acids (Asp881 and Glu882) due to the lack of electron density in the 2mFo‐DFc maps. Regarding the FAD binding site and the two [2Fe–2S] centres, no conformational changes were observed for these cofactors or corresponding domains.

### Thermostability of hAOX1 variants

**Figure 2:**
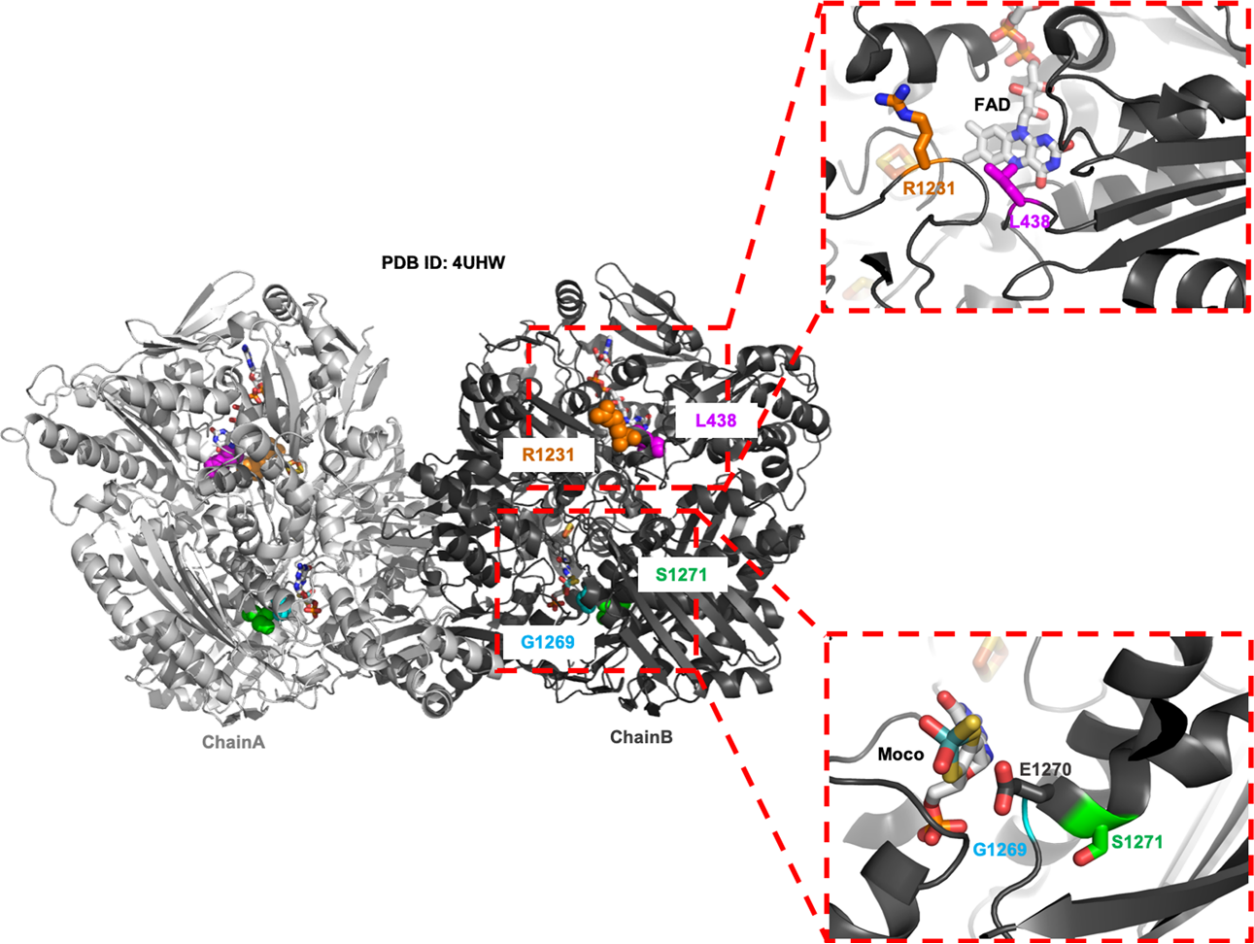
Overall structure of hAOX1 with location of the four SNPs of this study: G1269R and S1271L close to the Moco active site (lower panel) and L438V and R1231H at the entrance of the FAD pocket (top panel).

The use of *in vitro* screening tools applied to AOX studies is of great interest and could be an asset to the drug development community. In this study, we employed two biophysical methods (CD spectroscopy and ThermoFAD) to investigate the stability of the natural variants L438V, R1231H, G1269R and S1271L (Fig. 2) analysed in comparison with the WT hAOX1.

**Figure 3:**
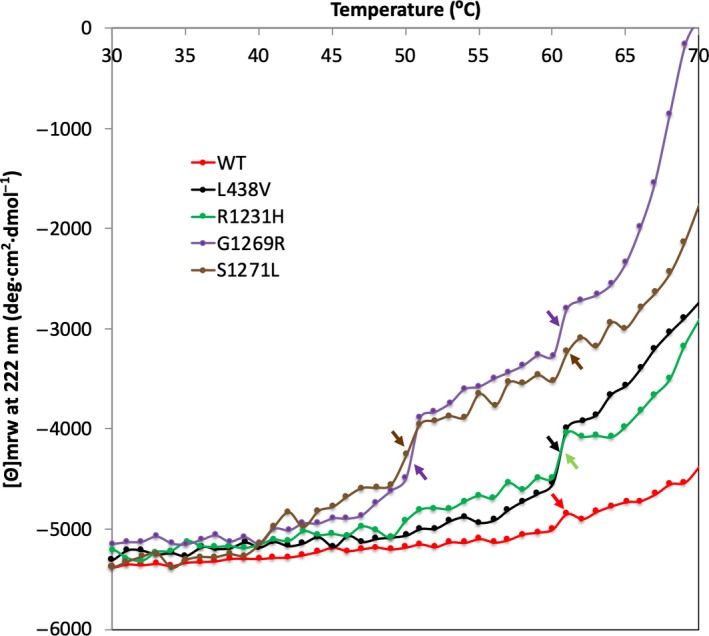
The denaturation curves of hAOX1 SNPs and WT using the loss of the CD signal at 222 nm while the temperature is increasing from 10 to 70 °C (only shown from 30 °C). One unfolding transition state around 60 °C is pronounced for R1231H and L438V similar to that observed for the WT. However, two jumps are visible for G1269R and S1271L variants that suggest less stability for the variants with amino acid changed around the Mo active site.

In general, CD spectroscopy is a technique widely used to study the secondary structures of proteins 23. In order to get reliable CD results, it is essential to have a homogenous sample with the protein concentration carefully determined 23. The active form of hAOX1 is the dimeric state and the main fraction in a protein sample 10, 12; however, we discovered that the tetramer, dimer and monomer states are in equilibrium in the solution in a concentration‐dependent manner. Such equilibration affects the amount of the secondary structure and the unfolding behaviour of each oligomeric sate. In this study, we were not looking for absolute secondary structures, but were following the changes of the secondary structure with the temperature in comparison with the hAOX1 WT protein. These direct comparisons with the WT under the same conditions gave us the possibility to apply CD measurements as an indicator of the stability of the hAOX1 SNP‐based variants. The melting curves obtained did not show ideal sigmoidal behaviour (Fig. 3), due to protein denaturation based on the high protein concentrations required for CD.

Therefore, we wanted to establish a novel method for the characterization of the protein stability of FAD‐containing enzymes. Thermofluor is a cheap and fast technique that constitutes an efficient and generic high‐throughput method for identification of protein properties being often used to optimize buffer and crystallization conditions, to find new ligands and to evaluate the impact of mutations in terms of protein stability 24, 25, 26, 27. In addition, this technique uses low amounts of sample when compared with CD spectroscopy (20 μL of 1 μm vs 400 μL of 40–45 μm protein). The methodology takes advantage of the fact that the fluorescence of many nonspecific protein‐binding dyes increases with increasing hydrophobicity of their environment 25. The fact that AOX binds a FAD cofactor enables it to be used as an intrinsic probe and monitor protein folding and stability, probing the different fluorescent properties between the folded and denatured state. This modified Thermofluor approach was designated ThermoFAD 28 and can be used in all flavin‐containing proteins. The main advantage of this method is that it allows a large amount of biochemical data to be obtained using very small amounts of protein sample and standard laboratory equipment without the requirement of an organic dye.

**Figure 4:**
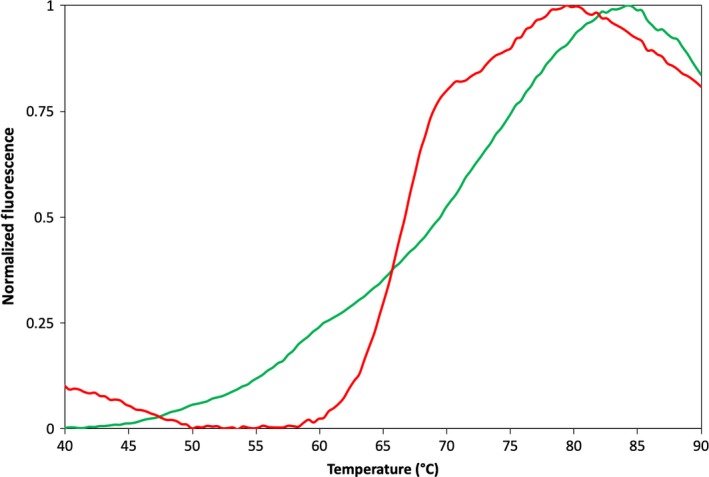
Comparison between Thermofluor and ThermoFAD data for hAOX1 WT. Thermal stability curves are plotted against normalized fluorescence signal. Green line, Thermofluor experiment using PTSD from Applied Biosystems as fluorescent probe; red line, ThermoFAD experiment measured without addition of any dye. Measurements were performed using an excitation wavelength of 470 nm and the ROX fluorescence emission filter (~ 610 nm).

In order to validate the ThermoFAD technique when applied to the hAOX1 protein, we compared the melting curves of the protein, in the presence (Thermofluor assay) and absence (ThermoFAD assay) of the protein thermal shift dye (PTSD) from Applied Biosystems (Waltham, MA, USA). As expected, the fluorescence signal intensity from the FAD cofactor is lower when compared to the signal from the PTSD (in the Thermofluor assay). This is mainly due to the much higher concentration of the PTSD compared to the natural fluorophore (FAD) and also to the fact that the experimental settings were optimized by the manufacturer for using PTSD. Nevertheless, the two melting curves were normalized and compared (Fig. 4), showing sigmoidal curves with slightly different calculated melting temperatures (*T*_m_) of 69.5 and 66.7 °C for Thermofluor and ThermoFAD assays, respectively. This difference can be explained either by the buffer conditions and the addition of an organic molecule (PTSD) that might stabilize the protein or by the fact that, while in the Thermofluor experiment the fluorescence corresponds to the denaturation of the entire protein, the ThermoFAD experiment targets the FAD molecule that is confined to a small area of the protein. However, the latest is unlikely since, as described below, modified residues located far from the FAD site have produced differences in the ThermoFAD melting curve, confirming that this experiment is valid to screen any modification in the protein, independent of its location.

In order to study the effect of missense SNPs on the stability of hAOX1, we compared WT hAOX1 with four SNPs characterized previously: S1271L and G1269R (crystal structure in this work) that are adjacent to the catalytic glutamate (Glu1270), and L438V and R1231H located in the proximity of the FAD cofactor (Fig. 2). We determined *T*_m_ using both ThermoFAD and CD spectroscopy techniques.

**Figure 5:**
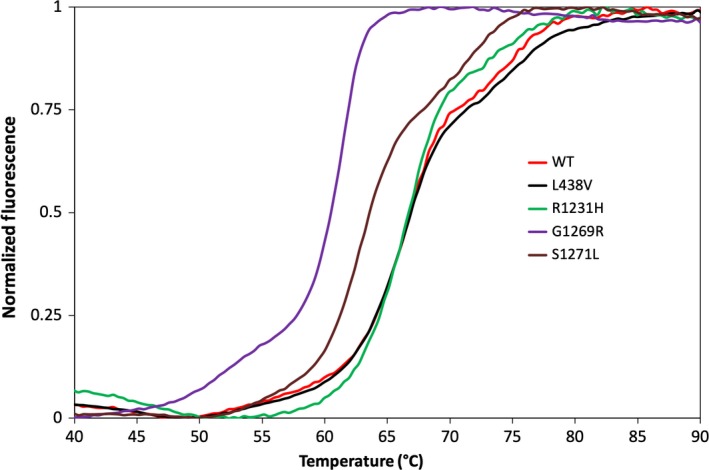
ThermoFAD of hAOX1 SNPs and WT. Thermal stability curves are plotted against normalized fluorescence signal. hAOX1 WT (red), L438V (black), R1231H (green), G1269R (purple) and S1271L (brown). Measurements were performed using an excitation wavelength of 470 nm and the SYBR Green fluorescence emission filter (~ 520 nm).

The L438V and R1231H SNPs showed no significant shifts in the *T*_m_ values regarding the WT protein, using either CD or ThermoFAD (with a Δ*T*_m_ below or equal to 0.09 °C), suggesting that these mutations have no impact on the stability of the protein (Fig. 5, Table 2). Analysing the hAOX1 crystal structure, we can see that Leu438 (Ile431 in bovine XO) is at 3.5 Å from the FAD isoalloazine ring, establishing a hydrophobic interaction (Fig. 2). Replacing this residue by the shorter, but also hydrophobic, side chain (Val) should have no major effect on this region stability or overall structure. However, this mutation affects the phenanthridine oxidation in a slightly positive fashion (Table 2) and increases the production rate of superoxide radical around 72% 15. In the case of the Arg1231, we also observed that the substitution of the arginine by a histidine did not show any significant protein stability differences, since this flexible residue is located in a solvent exposed region, with poorly defined electron density (PDB ID: 4UHW). Nevertheless, R1231H shows a decreased activity (Table 2), which may be due to changes in the electrostatic environment at the FAD site.

On the other hand, the G1269R and S1271L variants that are buried residues close to the Mo site present considerable shifts in ThermoFAD denaturation curves, with lower *T*_m_ values of 60.4 and 63.6 °C, respectively (Fig. 5, Table 2). The CD denaturation curves (Fig. 3) showed two unfolding states for these two variants. The higher *T*_m_ (60.03 °C and 60.13 °C for G1269R and S1271L, respectively) appeared at similar temperatures as hAOX1 WT, while L438V and R1231H were denatured with more pronounced loss of the CD signal. This corresponds to the global denaturation and total loss of secondary structure that occurs more drastically for G1269R and S1271L. In addition, these two variants showed an initial unfolding state at 50.05 °C and 50.04 °C (Fig. 3 and Table 2), respectively. This may be due to the Moco domain denaturation that unfolds, at least partially, before the global unfolding occurs and shows the effect of Moco incorporation on the stability.

The results suggest that the amino acid exchanges in the variants G1269R and S1271L have affected the stability of the protein. In fact, the G1269R variant corresponds to a Moco‐free form of the enzyme where the whole cofactor is absent, as reported here, and, as expected, this variant has no measurable activity 16. In contrast, the structure of the S1271L variant previously reported (PDB ID: 5EPG) 16 did not show any significant structural differences when compared to the WT enzyme. Nevertheless, the replacement of a polar amino acid (Ser) by a hydrophobic residue (Leu) in the core of the protein that is surrounded by a hydrophobic neighbourhood affects the protein dynamics. This may suggest that, although the global protein structure is not greatly affected at cryogenic temperature, the substitution of a serine by a leucine residue might affect the flexibility of the Moco domain (not evident on the crystal structure). In addition, the catalytic efficiency of S1271L seems to decrease when compared to the WT hAOX1 16.

## Conclusions

We have solved the first crystal structure of the natural variant G1269R of hAOX1 that was revealed to be a Moco‐free form of the enzyme. As in the case of the available Moco‐free crystal structures of rat liver XO, no major changes were observed in the overall structure of the enzyme. Nevertheless, in G1269R, important structural modifications were observed in the active site, with the rearrangement of Ser1986‐Gly‐Gly‐Ser1089 loop and the presence of Arg1269 at the active site pocket, which has prevented the insertion of the Moco cofactor into the protein. These results have important implications to the study of the Moco cofactor insertion, which has always been a puzzling question. The cofactor is deeply buried within the holoenzyme, and it would be expected that the protein could not fold properly without it. However, the structure of a Moco‐free form shows a network of hydrogen bonds able to preserve the protein architecture even in its apo form. This raises the possibility that the enzyme maturation and Moco insertion may occur into the properly folded apo‐enzyme.

Additionally, this Moco‐free crystal structure of hAOX1 allowed us to trace for the first time the six amino acids constituting the flexible Gate 1 that conditions the access into the Mo site. Together with the WT structure (PDB ID: 4UHW), the protein model is now more accurate and suitable for posterior analysis, namely molecular dynamics or docking experiments.

Finally, in this study we were also able to establish a simple, reliable and reproducible ThermoFAD technique to analyse the impact of the different hAOX1 SNPs on the stability of the enzyme. The ThermoFAD results were compared to the Tms obtained from the well‐established thermal denaturation procedure by CD spectroscopy. Despite the differences in the principles of these two techniques, both provided comparable data to report the protein stability. ThermoFAD showed to be a simpler technique to study the complex AOX system and, additionally, is a high‐throughput technique that requires low amounts of protein. These stability assays correlate structural and biochemical data of the different SNPs with the location of the changed amino acids. It seems that the changes around the Moco active site have a more pronounced effect on the protein stability with the Moco‐free variant being the less stable of all the variants. This may also imply that the properly inserted Moco also confers thermostability to the corresponding enzymes.

The ensemble of these studies shall contribute to a better understanding of hAOX1 and to the study of the effect of SNPs in the human population.

## Methods

### Enzyme production and characterization

Human aldehyde oxidase 1 WT and enzyme variants L438V, R1231H, G1269R and S1271L were expressed in an *Escherichia coli* expression system and purified as described previously 16, 17.

### Crystallization and data collection

Human aldehyde oxidase 1‐G1269R was pre‐incubated with 30 mm of DTT before setting up the crystallization drops. The crystals of the G1269R variant were grown in hanging drops at 293 K, using 0.1 m sodium malonate (pH 5.0) and 20% poly(ethylene glycol) 3350 as precipitant solution. Star‐shaped crystals appeared a few hours after setting up the crystallization drops and grew to their maximum size (0.1 mm length) within 24 h. They were rapidly transferred into a cryoprotectant solution consisting of the reservoir solution with 15% (v/v) glycerol and were flash‐cooled in liquid nitrogen (100 K). The G1269R crystals diffracted up to 3.1 Å resolution at beamline ID30B 29 from the European Synchrotron Radiation Facility (ESRF, Grenoble, France) at 0.976 Å wavelength. The crystals belong to space group *P4*_*2*_ *2*_*1*_ *2*, with unit cell dimensions *a *= *b *=* *148.20 Å and *c *=* *132.20 Å. The collected X‐ray diffraction data sets were processed using the software packages XDS 30 and Aimless 31. Data collection statistics are presented in Table 1.

**Table 1:** Data collection and refinement statistics of hAOX1‐G1269R

Statistics	hAOX1‐G1269R
Data collection	
X‐ray source	ID30B (ESRF)
Wavelength (Å)	0.976
Resolution limit (Å)	49.40–3.10 (3.29–3.10)
Space group	*P4*_*2*_ *2*_*1*_ *2*
Cell parameters	
*a* = *b* (Å)	148.20
*c* (Å)	132.20
Total no. of reflections	230 122 (37 789)
No. of unique reflections	27 261 (4329)
Completeness (%)	99.9 (100.0)
Redundancy	8.4 (8.7)
*R*_merge_ (%)	10.3 (86.3)
CC1/2	0.999 (0.728)
*<I/*σ(*I*)>	15.6 (2.4)
Refinement	
Resolution range (Å)	46.86–3.10
*R*_work_ (%)	19.16
*R*_free_ (%)	23.57
RMSD from ideal geometry	
Bonds (Å)	0.007
Angles (°)	0.496
Avg *B* factor (Å^2^)	
Protein	79.8
Cofactors	65.0
Ligands	83.2
Waters	62.2
Ramachandran plot (%)	
Favoured	95.85
Allowed	4.07
Outliers	0.08

### Structure solution, model building and refinement

The G1269R structure was solved by molecular replacement with the program Phaser 32, using the structure of the hAOX1‐free protein as a search model (PDB ID: 4UHW). Manual model building and iterative refinement were carried out using Coot 33 and phenix.refine 34. The refinement was conducted with secondary structure restraints. The quality of the model was confirmed by MolProbity 35. The final protein model includes residues 4‐1336, excluding the gaps 168–198, 558, 570–571, 714–715, 881–882 and 1256–1258 due to the lack of electron density to fit the main chain, two [2Fe‐2S] clusters, one FAD, 16 waters and 4 malonate molecules. The final *R*_work_ and *R*_free_ values were 19.16 and 23.57, respectively. Refinement statistics are shown in Table 1. The coordinates were deposited in the PDB under the accession code 6Q6Q.

### CD spectroscopy

The CD temperature‐dependent experiments were performed using a JASCO 815 CD spectropolarimeter equipped with a Peltier PTC‐423S device to maintain the temperature within ± 0.1 °C. Each sample contained 400 μL of protein solution in a concentration range between 40 and 45 μm in buffer (50 mm Tris/HCl, 1 mm EDTA, 200 mm NaCl, pH 8.0). CD spectra for each sample were recorded in a quartz cuvette of 1 mm path length (Hellma, Germany). The instrument was calibrated using 1S‐(+)‐10‐camphorsulphonic acid 23. After baseline correction, measured ellipticities were converted into mean residue ellipticities [ϴ]_MRW_. To follow protein unfolding, the CD signal at 222 nm was recorded while increasing the temperature from 10 to 70 °C with a ramp of 1 °C per minute. Similar to measurements of full spectra, the data were baseline‐corrected and converted to [ϴ]_MRW_. Final curves were normalized to the WT values for better comparison. All measurements were repeated two times. The first derivatives of the denaturation curves were obtained, and the global minima were considered as the *T*_m_ summarized in Table 2.

**Table 2:** Calculated melting temperatures and catalytic activities for WT and each SNP. The results are means ± SD of independent results

hAOX1 protein	Domain location of the variant	ThermoFAD		CD spectroscopy		Activity (%)^a^	PDB ID
		*T*_m_ (°C)	Δ*T*_m_ (°C)	*T*_m_ (°C)	Δ*T*_m_ (°C)		
WT	–	66.80 (± 0.06)	–	60.03 (± 0.02)	–	100 15, 16	4UHW 10
L438V	FAD vicinity	66.82 (± 0.09)	0.02	60.03 (± 0.02)	0	126 15	–
R1231H	FAD vicinity	66.71 (± 0.02)	−0.09	60.06 (± 0.01)	0.03	48 15	–
S1271L	Moco vicinity	63.55 (± 0.03)	−3.25	60.13 (± 0.04)	0.10	76 16	5EPG 16
				50.04 (± 0.01)	−10.09		
G1269R	Moco vicinity	60.44 (± 0.05)	−6.36	60.03 (± 0.01)	0	0 16	6Q6Q
				50.05 (± 0.07)	−10.08		

a *k*_cat_ values of hAOX WT in 15, 16 were attributed as 100% and used to determine the relative activity. The kinetic assays used phenanthridine as substrate and molecular oxygen as electron acceptor.

### Thermofluor and ThermoFAD assays

The Thermofluor and ThermoFAD assays were performed using the StepOnePlus System from Applied Biosystems, and the measurements were done using an excitation wavelength of 470 nm. The Thermofluor set‐up used the ROX fluorescence emission filter (~ 610 nm), while the ThermoFAD used the SYBR Green fluorescence emission filter (~ 520 nm). Thermofluor reactions were prepared using the PTSD Kit from Applied Biosystems, and the protein was diluted to a final concentration of 1 μm in 20 μL of reaction volume. In ThermoFAD experiments, the protein concentration required for optimal signal‐to‐noise ratio was 5 μm, and the reaction buffer used was 50 mm Tris/HCl (pH 8.0):200 mm NaCl in a final reaction volume of 20 μL. Unfolding curves were generated using a temperature gradient from 25 to 95 °C in 46 min. All experiments were performed in triplicate, and the reported *T*_m_ values are based on the mean values determined from the minimum value of the inverse of the first derivative of the experimental data.

## Conflict of interest

The authors declare no conflict of interest.

## Author contributions

CM, CC, TSS and MJR conceived and designed the project; CM, ME, MW and AF acquired the data; CM, ME, CC, TSS, MW, SL and MJR analysed and interpreted the data; and CM, ME, CC, TSS, SL and MJR wrote the paper.
